# Supplementary material for: SARS-CoV-2 vaccination, booster, and infection in pregnant population enhances passive immunity in neonates
Source: Nat Commun. 2023 Aug 10;14:4598. doi: 10.1038/s41467-023-39989-y (PMC10415289; doi:10.1038/s41467-023-39989-y)
Supplement: Supplementary file 1 — Supplementary Information [file 41467_2023_39989_MOESM1_ESM.pdf]

Supplementary Table 1: Demographics, Vaccination Data, and History of Infection on complete cohort stratified by vaccination status and infection history

	Total Population	NoVx	PartVx	FullVx	BoostVx	NoVx Inf	PartVx Inf	FullVx inf	BoostVx Inf
Patient samples, n	4600	1601	194	1311	483	508	51	267	185
Age, Median (IQR) years	35 (6)	35 (6)	36 (5)	35 (5)	35 (5)	33 (6)	35 (6)	35 (5)	35 (5)
Gestation age at delivery, Median (IQR) weeks	39.3 (1.50)	40.4 (0.65)	39.3 (1.55)	39.3 (1.4)	39.2 (1.7)	35.8 (1.65)	38.9 (1.8)	39.5 (1.55)	39.3 (1.4)
Umbilical Cord Blood Samples captured, n	2706	28	186	1284	471	249	52	262	174
Vaccination Course received, n									
Pfizer-BioNTech	1800	---	141	975	336	---	37	186	125
Moderna	616	---	52	303	127	---	13	67	54
Johnson & Johnson	64	---	1	32	16	---	1	10	4
Mixed dosing	13	---	0	1	4	---	0	4	2
None	2109	1601	---	---	---	508	---	---	---
Booster Vaccine received, n									
Pfizer	481	---	---	---	349	---	---	---	132
Moderna	186	---	---	---	134	---	---	---	52
Johnson & Johnson	1	---	---	---	0	---	---	---	1
None	2331	1601	194	1311	---	508	51	267	---
Gestational age at first vaccination dose, n									
Before pregnancy	719	---	2	92	355	---	5	90	175
1st trimester	508	---	9	345	119	---	0	30	5
2nd trimester	704	---	5	582	3	---	15	97	2
3rd trimester	545	---	178	287	1	---	31	48	0
Unknown		---	0	5	5	---	0	2	3
Gestational age at second vaccination dose, if received, n									
Before pregnancy	590	---	0	48	307	---	0	70	165
1st trimester	387	---	0	201	145	---	0	28	13
2nd trimester	674	---	0	586	9	---	0	79	0
3rd trimester	689	---	141	444	0	---	24	80	0
Unknown		---	0	0	22	---	0	10	7
Gestational age at third vaccination dose, if received, n									
Before pregnancy	3	---	---	---	1	---	---	---	2
1st trimester	24	---	---	---	18	---	---	---	6
2nd trimester	317	---	---	---	205	---	---	---	112
3rd trimester	324	---	---	---	259	---	---	---	65

Supplementary Table 1: Full course of vaccination was categorized as 1 dose for Johnson &Johnson vaccination, 2 doses for Pfizer-BioNTech vaccination, and 2 doses for Moderna vaccination. Patients were listed as not having received any vaccination (NoVx), started a vaccination course but not yet 14 days post the completion of a full course of vaccination (PartVx), at least 14 days post completion of a full course of vaccination (FullVx), or received an additional booster dose after completion of a full course of vaccination (for a total of 2 doses for Johnson&Johnson, 3 doses for Pfizer-BioNTech, 3 doses for Moderna) (BoostVx). The BoostVx patients were confirmed to not have any immunosuppressing condition or use of an immunosuppressing medication. Mixed dosing refers to patients that received a combination of Pfizer/BioNTech and Moderna for the vaccination course. Patient counts are stratified according to vaccination status at delivery and positive history of infection (Inf).

Supplementary Table 2: Demographics, Vaccination Data, and History of Infection on cohort of patients that neutralization studies were performed.

Patient samples, n	259
Age, Median (IQR) years	34 (6)
Gestation age at delivery, Median (IQR) weeks	39.3 (1.7)
Umbilical Cord Blood Samples captured, n	260
Vaccination Cohorts, n	
No History of infection	
NoVx	--
PartVx	--
FullVx	--
BoostVx	--
Positive History of Infection	
NoVx/Inf	77
PartVx/Inf	10
FullVx/Inf	59
BoostVx/Inf	113
Primary Vaccination Course received, n	
Pfizer-BioNTech	119
Moderna	53
Johnson & Johnson	7
Mixed dosing	3
Booster Vaccine received, n	
Pfizer-BioNTech	82
Moderna	31
Johnson & Johnson	--
Gestational age at first vaccination dose, n	
Before pregnancy	154
1st trimester	9
2nd trimester	12
3rd trimester	4
Gestational age at second vaccination dose, n	
Before pregnancy	138
1st trimester	15
2nd trimester	9
3rd trimester	2
Gestational age at third vaccination dose, if received, n	
Before pregnancy	1
1st trimester	2
2nd trimester	72
3rd trimester	38

Supplementary Table 2: Neutralization studies were performed on a representative cohort of 259 patients and corresponding 260 neonates with positive history of infection and who had enough specimen leftover to perform neutralization studies on the same sample. This cohort was also selected based on the documentation of a specific date of COVID-19 infection diagnosis. Full course of vaccination was categorized as 1 dose for Johnson &Johnson vaccination, 2 doses for Pfizer-BioNTech vaccination, and 2 doses for Moderna vaccination. Patients were listed as not having received any vaccination (NoVx), started a vaccination course but not yet 14 days post the completion of a full course of vaccination (PartVx), at least 14 days post completion of a full course of vaccination (FullVx), or received an additional booster dose after completion of a full course of vaccination (for a total of 2 doses for Johnson&Johnson, 3 doses for

Pfizer-BioNTech, 3 doses for Moderna) (BoostVx). The BoostVx patients were confirmed to not have any immunosuppressing condition or use of an immunosuppressing medication. Mixed dosing refers to patients that received a combination of Pfizer/BioNTech and Moderna for the vaccination course.

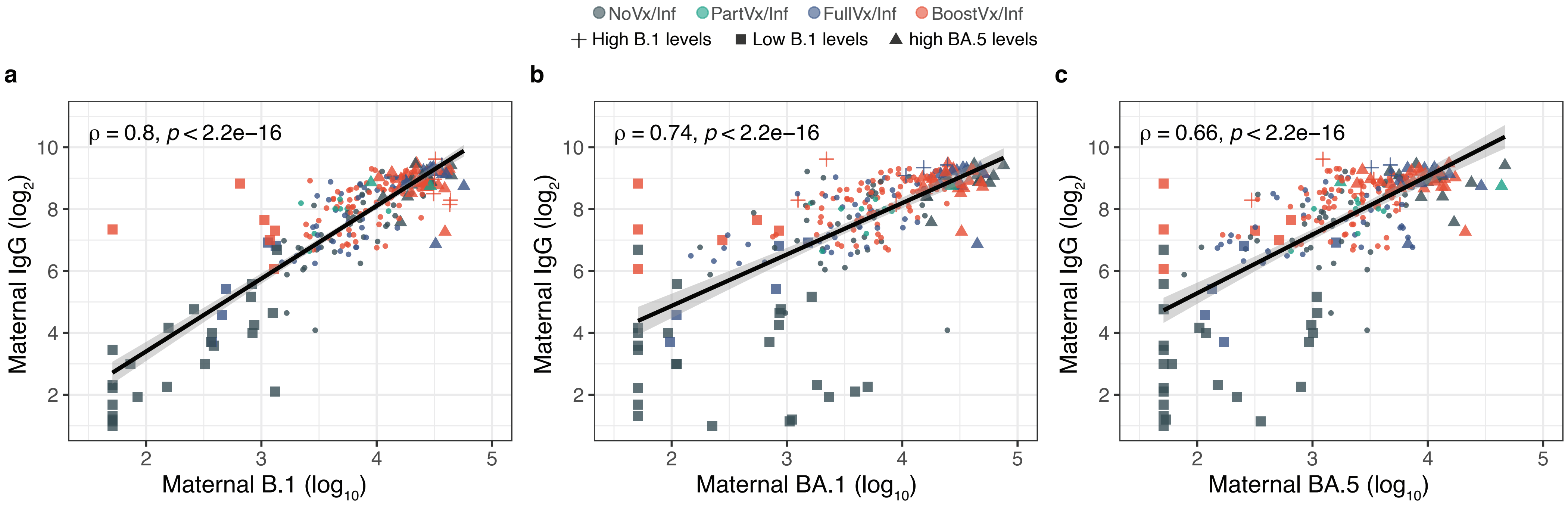
NoVx/Inf
PartVx/Inf
FullVx/Inf
BoostVx/Inf
High B.1 levels
Low B.1 levels
high BA.5 levels
a
b
c
ρ = 0.8, p < 2.2e−16
ρ = 0.74, p < 2.2e−16
ρ = 0.66, p < 2.2e−16
Maternal IgG (log2)
Maternal B.1 (log10)
Maternal BA.1 (log10)
Maternal BA.5 (log10)

Supplementary Figure Legends

Supplementary Fig. 1: Maternal neutralizing activity correlates with maternal anti-S IgG levels in patients with positive history of SARS-CoV-2 infection

a-c. Correlation between maternal anti-S IgG levels (log2 transformed) and neutralizing antibody levels (log10 transformed) in patients with positive history of SARS-CoV-2 infection. Not vaccinated with positive history of infection (NoVx/Inf, dark grey); Partially vaccinated with positive history of infection (PartVx/Inf, dark green); Fully vaccinated with positive history of infection (FullVx/Inf, dark blue); Boosted with positive history of infection (BoostVx/Inf, dark red). Spearman correlation analysis were carried out for each variant assayed: B.1; BA.1; BA.5. Patients with high B.1 levels are depicted with a plus sign; patients with low B.1 levels are depicted as a square; patients with high BA.5 levels are shown via triangle; all remaining patients are represented as a circle. The half-maximal neutralization titers for each plasma (NT50).
