# Supplementary material for: SARS-CoV-2 vaccination, booster, and infection in pregnant population enhances passive immunity in neonates
Source: Nat Commun. 2023 Aug 10;14:4598. doi: 10.1038/s41467-023-39989-y (PMC10415289; doi:10.1038/s41467-023-39989-y)
Supplement: Supplementary file 3 — Reporting Summary [file 41467_2023_39989_MOESM3_ESM.pdf]

nature portfolio

Corresponding author(s): Yawei Jenny Yang
Theodora Hatziioannou

Last updated by author(s): Jun 28, 2023

Reporting Summary

Nature Portfolio wishes to improve the reproducibility of the work that we publish. This form provides structure for consistency and transparency in reporting. For further information on Nature Portfolio policies, see our Editorial Policies and the Editorial Policy Checklist.

Statistics

For all statistical analyses, confirm that the following items are present in the figure legend, table legend, main text, or Methods section.

n/a	Confirmed	
☐	☒	The exact sample size (n) for each experimental group/condition, given as a discrete number and unit of measurement
☐	☒	A statement on whether measurements were taken from distinct samples or whether the same sample was measured repeatedly
☐	☒	The statistical test(s) used AND whether they are one- or two-sided<br>*Only common tests should be described solely by name; describe more complex techniques in the Methods section.*
☐	☒	A description of all covariates tested
☐	☒	A description of any assumptions or corrections, such as tests of normality and adjustment for multiple comparisons
☐	☒	A full description of the statistical parameters including central tendency (e.g. means) or other basic estimates (e.g. regression coefficient) AND variation (e.g. standard deviation) or associated estimates of uncertainty (e.g. confidence intervals)
☐	☒	For null hypothesis testing, the test statistic (e.g. F, t, r) with confidence intervals, effect sizes, degrees of freedom and P value noted<br>*Give P values as exact values whenever suitable.*
☒	☐	For Bayesian analysis, information on the choice of priors and Markov chain Monte Carlo settings
☒	☐	For hierarchical and complex designs, identification of the appropriate level for tests and full reporting of outcomes
☐	☒	Estimates of effect sizes (e.g. Cohen's d, Pearson's r), indicating how they were calculated

Our web collection on statistics for biologists contains articles on many of the points above.

Software and code

Policy information about availability of computer code

Data collection	GraphPad Prism version 8.0; R version 4.1.0 (2021-05-18), RStudio 1.1.463
Data analysis	All analyses were performed using R Statistical Software (v4.1.0; R Core Team 2021) and RStudio 1.1.463

For manuscripts utilizing custom algorithms or software that are central to the research but not yet described in published literature, software must be made available to editors and reviewers. We strongly encourage code deposition in a community repository (e.g. GitHub). See the Nature Portfolio guidelines for submitting code & software for further information.

Data

Policy information about availability of data

All manuscripts must include a data availability statement. This statement should provide the following information, where applicable:

- Accession codes, unique identifiers, or web links for publicly available datasets
- A description of any restrictions on data availability
- For clinical datasets or third party data, please ensure that the statement adheres to our policy

All data generated in this study are provided in the Source Data file.

Research involving human participants, their data, or biological material

Policy information about studies with human participants or human data. See also policy information about sex, gender (identity/presentation), and sexual orientation and race, ethnicity and racism.

Reporting on sex and gender	This study included 4600 reproductive aged females. The findings in the study apply only to the female sex. Gender was not collected from participants and was therefore not considered in the study design.
Reporting on race, ethnicity, or other socially relevant groupings	Patient demographic (race, ethnicity) data was not available and was not included in this study.
Population characteristics	Our study included 4600 pregnant patients who gave birth at an academic medical center in New York City between April 18, 2020, and April 27, 2022. The median (IQR) maternal age at the time of delivery was 35 (6) years and the median (IQR) gestational age at delivery was 39.3 (1.50) weeks
Recruitment	Patients that delivered at an academic medical center in New York City between April 18, 2020, and April 27, 2022, were included in this study. Patients were included if they had documented receipt of at least one dose of a SARS-CoV-2 vaccine (Pfizer-BioNTech, Moderna, or Johnson&Johnson) or a history of SARS-CoV-2 infection.
Ethics oversight	This research complies with all relevant ethical regulations and was approved by the Weill Cornell Medicine Institutional Review Board. A waiver of informed consent was granted by the Weill Cornell Medicine Institutional Review Board.

Note that full information on the approval of the study protocol must also be provided in the manuscript.

Field-specific reporting

Please select the one below that is the best fit for your research. If you are not sure, read the appropriate sections before making your selection.

☒ Life sciences ☐ Behavioural & social sciences ☐ Ecological, evolutionary & environmental sciences

For a reference copy of the document with all sections, see nature.com/documents/nr-reporting-summary-flat.pdf

Life sciences study design

All studies must disclose on these points even when the disclosure is negative.

Sample size	No sample size calculation was performed, sample size was determined based on patient volume during time of study April 18, 2020, and April 27, 2022.
Data exclusions	No data were excluded from the analyses.
Replication	Earlier Small scale analysis results were matched and reproduced with larger cohort. Results matched similar patterns in previous cohorts.
Randomization	Patients were not randomly allocated into groups. Groups were determined based on patient clinical history and vaccination status at time of delivery.
Blinding	Investigators were blinded to group allocation during data collection since the clinical data was integrated at the analysis step. Investigators were not blinded to group allocation during data analysis.

Reporting for specific materials, systems and methods

We require information from authors about some types of materials, experimental systems and methods used in many studies. Here, indicate whether each material, system or method listed is relevant to your study. If you are not sure if a list item applies to your research, read the appropriate section before selecting a response.

Materials & experimental systems

n/a	Involved in the study	
☐	☐	Antibodies
☐	☒	Eukaryotic cell lines
☒	☐	Palaeontology and archaeology
☒	☐	Animals and other organisms
☒	☐	Clinical data
☒	☐	Dual use research of concern
☒	☐	Plants

Methods

n/a	Involved in the study	
☒	☐	ChIP-seq
☒	☐	Flow cytometry
☒	☐	MRI-based neuroimaging

Antibodies

Antibodies used	*Describe all antibodies used in the study; as applicable, provide supplier name, catalog number, clone name, and lot number.*
Validation	*Describe the validation of each primary antibody for the species and application, noting any validation statements on the manufacturer's website, relevant citations, antibody profiles in online databases, or data provided in the manuscript.*

Eukaryotic cell lines

Policy information about cell lines and Sex and Gender in Research

Cell line source(s)	293T (ATCC, CRL-11268) and HT1080 (ATCC, CCL-121)
Authentication	ATCC performs authentication and quality-control tests on all cell lines distributed. None of the cell lines used were authenticated after purchase.
Mycoplasma contamination	Cells were periodically checked for mycoplasma and retrovirus contamination by DAPI staining and reverse transcriptase assays, respectively.
Commonly misidentified lines (See ICLAC register)	No commonly misidentified cell lines were used in the study.
